# Understanding factors influencing linkage to HIV care in a rural setting, Mbeya, Tanzania: qualitative findings of a mixed methods study

**DOI:** 10.1186/s12889-019-6691-7

**Published:** 2019-04-05

**Authors:** Erica S. Sanga, Ferdinand C. Mukumbang, Adiel K. Mushi, Wondwossen Lerebo, Christina Zarowsky

**Affiliations:** 1 NIMR-Mwanza Medical Research Centre, P.O Box 1462, Mwanza, Tanzania; 2 School of Public Health- University of Western Cape, Cape Town, South Africa; 3 School of Public Health, Mekelle University, Makelle, Ethiopia; 4 National Institute for Medical Research (NIMR), London, England; 5 University of Montreal Hospital Research Centre and School of Public Health, Universite de Montreal, Montreal, Canada; 6 Department of Public Health, Institute of Tropical Medicine, Antwerp, Belgium; 7 NIMR-Mwanza Medical Research Centre (MMRC), Mbeya, Tanzania

**Keywords:** HIV, Linkage to care, Facilitators to HIV care linkage, Barriers to HIV care linkage, Qualitative research, Tanzania, Health system, Quality of care

## Abstract

**Background:**

In remote rural Tanzania, the rate of linkage into HIV care was estimated at 28% in 2014. This study explored facilitators and barriers to linkage to HIV care at individual/patient, health care provider, health system, and contextual levels to inform eventual design of interventions to improve linkage to HIV care.

**Methods:**

We conducted a descriptive qualitative study nested in a cohort study of 1012 newly diagnosed HIV-positive individuals in Mbeya region between August 2014 and July 2015. We conducted 8 focus group discussions and 10 in-depth interviews with recently diagnosed HIV-positive individuals and 20 individual interviews with healthcare providers. Transcripts were analyzed inductively using thematic content analysis. The emergent themes were then deductively fitted into the four level ecological model.

**Results:**

We identified multiple factors influencing linkage to care. HIV status disclosure, support from family/relatives and having symptoms of disease were reported to facilitate linkage at the individual level. Fear of stigma, lack of disclosure, denial and being asymptomatic, belief in witchcraft and spiritual beliefs were barriers identified at individual’s level. At providers’ level; support and good patient-staff relationship facilitated linkage, while negative attitudes and abusive language were reported barriers to successful linkage. Clear referral procedures and well-organized clinic procedures were system-level facilitators, whereas poorly organized clinic procedures and visit schedules, overcrowding, long waiting times and lack of resources were reported barriers. Distance and transport costs to HIV care centers were important contextual factors influencing linkage to care.

**Conclusion:**

Linkage to HIV care is an important step towards proper management of HIV. We found that access and linkage to care are influenced positively and negatively at all levels, however, the individual-level and health system-level factors were most prominent in this setting. Interventions must address issues around stigma, denial and inadequate awareness of the value of early linkage to care, and improve the capacity of HIV treatment/care clinics to implement quality care, particularly in light of adopting the ‘Test and Treat’ model of HIV treatment and care recommended by the World Health Organization.

## Background

Sub-Saharan Africa (SSA) bears the highest HIV/AIDS burden in the world having an estimated 71% (25.8 million) of all people living with HIV in 2014 [1]. HIV is one of Tanzania’s major public health problems. An estimated 1.5 million people are living with HIV, representing a prevalence rate of 5.1% [1, 2]. The rates of HIV testing are low, particularly in rural areas [3, 4].

In 2015, the Ministry of Health and Social Welfare in Tanzania, in collaboration with other stakeholders, drafted the National Comprehensive HIV Testing and Counseling (HTC) guideline, which combines all implementation approaches to HTC into one document. The aim of this guideline was to improve HIV testing, HIV prevention, linkage and enrolment into care, retention and adherence in general HIV care and treatment, and management of comorbidities in HIV/AIDS.

In Tanzania, home-based and other outreach HIV testing programs have been put in place to improve the low uptake by community members of HIV testing and to better link those individuals who test positive to HIV care and treatment centers (CTCs). By increasing the types and number of HIV/AIDS treatment and care strategies and service outlets, the Ministry expected that successful linkage to HIV treatment and care among people living with HIV in Tanzania would improve [2].

Despite increasing rates of HIV testing following the expansion of outreach HIV testing approaches [5], studies conducted in various regions of Tanzania have reported low linkage to care for individuals who test HIV positive [6]. A study conducted in Mwanza (Northern Tanzania) reported a linkage of as low as 14% in the first 4 months after diagnosis [3]. While improvements are occurring, linkage to care remains poor. According to Simmelink [4], Ifakara, Tanzania has a linkage rate of 23.1%. Similarly, findings from the Mbeya Regional AIDS control program 2014 report show a linkage rate of 28%. These rates of linkage to HIV care from various regions in Tanzania are indicative of a generalized problem faced by the Tanzanian HIV and AIDS treatment implementation programs.

### Linkage to care: a crucial step in HIV control and disease outcome

Timely and effective linkage to HIV treatment, care and support are crucial for a better prognosis of HIV/AIDS. Hence, the most recent WHO “test-and-treat” guidelines for HIV treatment and care recommend that treatment should be immediately initiated once someone has tested positive for HIV, irrespective of their CD4 count [7]. Treatment initiation, nevertheless, can only be achieved if the HIV-positive individual is successfully linked to an accredited HIV treatment and care center or antiretroviral treatment scheme. As part of HIV/AIDS management implementation strategy, Tanzania’s National HIV testing and counseling guidelines (2013) stipulate that after completion of HIV testing, all HIV-positive individuals should be linked to receive appropriate care and treatment care services at designated care and treatment centers (CTCs) [8, 9].

Linkage to care is described as the process (interventions and programs) put in place to ensure that HIV-positive individuals are successfully entered into HIV medical care, psychological and social services [10–12]. The process of linkage to care includes educating patients about the benefits of being in care and providing facilitating services such as referral letters and guidance in selecting a treatment center and treatment options [13].

A range of factors may facilitate or mitigate the process of linkage in the trajectory of HIV care from the point of diagnosis to initiation of ART. These influences may occur at the level of the patient/individual, the health care provider, the health system, or may be influenced by other factors acting a structural or contextual level.

Several studies have explored the barriers and facilitating factors to linkage to care in Kenya, Uganda, Malawi and South Africa [14–19]. Fear of stigma, lack of disclosure of HIV status to relatives or significant others, being asymptomatic at the time of diagnosis and negative health care provider attitudes are some of the factors reported as barriers to timely linkage into HIV care [15, 20, 21]. Factors identified as facilitating linkage were the integration of HIV testing and HIV care services, good patient-staff relationships and short clinic waiting times [18, 20].

In Tanzania, Layer et al. [21] explored and classified the facilitating factors and barriers to linkage to HIV care and treatment services. Nevertheless, the literature specifically focusing on remote areas and hard-to-reach populations is scarce. In particular, we did not find any studies in the Mbeya region of Tanzania where population mobility and remoteness make linkage into HIV care particularly challenging [22].

To help fill this evidence gap, we conducted a longitudinal mixed methods study based on a two-armed cohort of 1012 newly HIV diagnosed individuals with a nested qualitative component in the main cohort. The first phase of the study compared rates and timelines of linkage to care between two models of HIV testing (mobile-based and facility-based) and found that at 6 months since diagnosis, 78% (793 out of 1012) of participants enrolled in the cohort had linked into care. Linkage to care was higher from facility-based than from mobile/outreach sites; 84% (CI = 81–87%, *n* = 512) of individuals tested at facility-based were linked to care, compared to 69% (CI = 65–74%, *n* = 281) of individuals tested at mobile/outreach in the same period. Similarly, Individuals tested at facility-based sites entered HIV care sooner than the individuals tested at the mobile/outreach sites. Disclosure of HIV status was a significant factor associated with timely linkage to care and clients who reported that wanting to get treatment as one of their reason for testing HIV were 25% more likely to link to care [23].

In the second phase, reported here, we aimed to describe the factors influencing linkage to HIV care services among newly diagnosed HIV-positive individuals in the mobile services and facility-based services, focusing on patient/individual, provider, health system and contextual levels, in order to contribute to a deeper, intervention-oriented understanding of the dynamics of linkage to care in Mbeya region, Southern highlands of Tanzania. We sought to examine and to inform the implementation strategies identified by Peters et al., [24] such as:

- Enhancing the capabilities of government (public policy and management oversight);
- Improving the performance of implementing and provider organizations;
- Strengthening the capabilities and performance of individual providers and front-line workers;
- Empowering communities, households and supporting multiple stakeholders engaged in improving health [24, 25]

### Study setting

The study was conducted in rural communities within Mbeya region, Southern Tanzania. This remote and transient region has a higher HIV prevalence than the national average (9% vs. 5.1%) and a high proportion of the population lives more than 10 km from a health facility (double the WHO recommended 5 km). The area is characterized by high rates of population mobility for cross-border trade between Tanzania, Zambia and Malawi. Both testing and linkage to care are particularly challenging in these circumstances.

The study took place in four purposively selected districts among the eight administrative districts of Mbeya region: Mbeya Rural, Kyela, Mbozi and Chunya. The selected districts included areas of high HIV prevalence and hard-to-reach populations. Two districts (Kyela and Mbozi) are along the highways and have borders with Zambia and/or Malawi. Mbeya Rural and Chunya districts have a larger proportion of residents who live 10 km or more from a health facility.

### Characteristics of health care provision in relation to HIV care in Mbeya region

In Mbeya region, about 23% of the inhabitants live within 5 km of a health facility, and 15% are more than 15 km away from a network of health facilities that include 20 hospitals, 36 health centers, and 374 dispensaries [26]. Out of these 490 health facilities, 350 facilities provide Prevention of Mother to Child Transmission (PMTCT) services, 312 provide HIV Counseling and Testing services while only 68 health facilities provide Antiretroviral therapy (ART) services [22]. The majority of health facilities in the rural areas do not offer CD4 testing or HIV treatment services although there has been a substantial increase in health facilities offering HIV testing and ART services in this region since 2010 [22].

### Design

The qualitative study reported here was nested within a larger mixed method cohort study that aimed to document and compare rates, patterns and determinants of linkage to care in the 6 months following an HIV positive test result between mobile and facility-based models of HIV testing in Mbeya region, Tanzania. The overall study design and methods are reported elsewhere [23].

We adopted a descriptive qualitative study design, an approach allowing for a ‘comprehensive summarisation of specific events experienced by individuals or groups of individuals’ [27]. We drew from the Ecological Model [28] to systematically explore the multi-level factors affecting linkage to HIV care. The ecological framework has been applied to explain HIV related treatment and care health seeking behavior using its proposed levels (1) intrapersonal or individual factors, (2) interpersonal factors, (3) institutional/ organizational factors (health system factors) (4) community factors or contextual factors. We found the Ecological Model useful as it assumes that behavior affects and is affected by multiple levels of influence [29]. On the basis of our experience in HIV intervention development, we felt that while this structured model may be less sensitive to complex findings than a fully inductive analysis, it is more accessible to and hence actionable by the decision makers and practitioners we sought to inform, whilst ensuring a comprehensive approach.

## Methods

Study participants were purposively selected among HIV-positive individuals who have been in the HIV care umbrella between 0 and 6 months. A total of 98 participants were included in the study. Sixty-eight HIV-positive individuals participated in eight focus group discussions (FGDs) each with six to 12 respondents. In-depth interviews were conducted (IDI’s) with another ten HIV-positive individuals. We also conducted 20 in-depth interviews with healthcare providers in the study sites, Table 1. The lead investigator (ES), assisted by four trained research assistants, conducted the interviews. We held a two-day training session on the data collection methods with the research assistants, before testing the data collection tools. We piloted the interviews and FGD guides in two sites (health facilities), which were not among the included districts research sites, and adjusted the instruments based on these pilots.

**Table 1:** Research sites and data collection techniques applied

Districts	Type of Site	No of FGDs	No of IDI-Clients	No of IDIs- HCP
Facility based sites				
Kyela	Kyela Hospital	0	1	2
	Ipinda Health Centre	1	1	1
Mbozi	Vwawa Hospital	0	0	1
	Tunduma health Centre	1	1	2
Mbeya Rural	Ifisi Hospital	1	1	1
	Inyala health centre	0	0	2
Chunya	Chunya hospital	1	1	2
	Makongolosi Dispensary	0	0	1
Mobile/outreach sites				
Kyela	ST JOHN HUS-Kyela	1	1	2
	MMRC mobile- Kyela	0	1	1
Mbozi	SHDEPHA - Mpemba	0	0	1
	MMRC Mobile-Mbozi	1	0	0
Mbeya Rural	KIHUMBE- Mbalizi	1	1	1
	MMRC mobile- Mbeya Rural	0	0	1
Chunya	KIHUMBE- Chunya	0	1	2
	MMRC- Mobile- Chunya	1	0	1
TOTAL		8	10	20

*FGD* Focus Group Discussions, *IDI* Individual Interviews, *HCP* Health Care Providers

The interview and focus group discussion guides were developed on the basis of the literature [14–19] and the responses to the questionnaire used in the earlier quantitative phase of the study. The interview guide for the patients focused on their experiences of being HIV positive, the circumstances around their registration into a treatment/care program and any challenges or enablers with regard to registration – considered the first step to linkage in care. The interview guide for the healthcare providers asked about the availability of HIV testing and linkage to care guidelines and whether these guidelines are being followed and explored the healthcare providers’ perspectives about what inhibits or enables individuals to link into care immediately after testing HIV positive.

Interviews and discussions were audio recorded and transcribed verbatim in Swahili. Two accredited translators translated the transcripts verbatim into English and the first author who is also fluent in both languages reviewed all translated transcripts. In analyzing the data, a thematic content analysis approach was used [25]. We applied deductive and inductive approaches in two phases. In phase one, we inductively coded the entire data set – making interpretations from the raw data. We analyzed and interpreted the data against the different levels of the ecological model and also included emerging themes from interviews.

We then fitted the identified codes within the ecological framework thus applying the deductive approach to thematic analysis. The coding of translated transcripts and organization of codes for deductive thematic content analysis was supported by Atlas.ti version 7 [30]. Finally, we included our earlier quantitative findings of the parent cohort study in the interpretation (presented in the Discussion section) to enrich the understanding of both quantitative and qualitative findings. A more fully integrated mixed methods analysis of all study findings is in preparation and not presented here.

### Rigor and trustworthiness

Following Lincoln and Guba [31] and Shenton [32], we employed several strategies to enhance credibility, transferability, dependability and confirmability. We adopted a qualitative method with three methods of data collection to explore and complement the findings of a quantitative study. Respondent validation by recapping the discussions with validation by the respondents was done during the focus group discussions. We applied different data collection techniques with a variety of respondents who were selected from multiple HIV testing sites.

We addressed dependability by training and supervision of a small number of field workers and by ensuring that interview and focused-group transcripts were translated from Swahili to English by two professional translators and checked by the first author who is fluent in both languages. Two authors analyzed the data and cross verified by the last author.

For confirmability, we kept an audit trail during the course of the study. Finally, we adopted the relevant guidelines of the 32-item checklist for reporting qualitative research (COREQ) as prescribed by Tong, Sainsbury, & Craig, [33] to report important aspects of the research team, study methods, context of the study, findings, analysis and interpretations.

### Ethical considerations

The study received ethical clearance from the following Ethics review boards: University of Western Cape (UWC) Senate Research Committee, Mbeya Medical Research Center, Mbeya Regional Medical Research Ethics Committee (MMREC) and the National Ethical Committee/ Medical Research Coordinating Committee at the National Institute for Medical Research in Tanzania.

Willing participants were provided with an information sheet detailing the nature, aim, and significance of the study. Participation was voluntary, and participants were advised that they were free to withdraw from the study at any time without negative consequences. Willingness to participate was confirmed by signing an informed consent form. Confidentiality and anonymity were considered by using pseudonyms and numbers to represent participants.

## Results

### Study sites and characteristics of respondents

A total of 38 males and 60 females above 18 years participated in qualitative interviews, with two respondents participating in both IDI and FGD.

### Factors identified as influencing linkage to care

Themes related to factors influencing linkage to care were categorized into four levels based on the conceptual framework used in the study: individual, health care provider, health system and contextual levels. Table 2 below illustrates the various categories into which the emerging themes and factors were classified. We present the identified factors affecting implementation at various levels first, reporting facilitators and then barriers, and then we report the most frequently mentioned factors from focus group discussions, individual patient interviews and individual health care provider interviews.

**Table 2:** Facilitators and barriers to linkage in care by levels

Level	Categories	Themes/Factors
Individual	Facilitators	Individuals being sick at the time of diagnosis
		Individuals disclosing their status to someone
		Social and moral support from family members/relatives and from other PLHIV
	Barriers	Fear of stigma
		Denial and being asymptomatic
		Poor health literacy
		Lack or fear of disclosure
		Belief in witchcraft and traditional treatment
		Spiritual beliefs
Health Care Provider	Facilitators	Support or encouragement from health care providers
		Good patient-healthcare provider relationship
	Barriers	Negative attitude from healthcare provider
Health System	Facilitators	Availability of referral procedures i.e. referral letter/Referral form
		Good service organization
	Barriers	Poor clinic procedures and visit schedules
		Clinic over-crowding
		Long waiting times at the clinic
		Few care and treatment centers and inadequate resources
		Shortage of staff
		Inadequate CD4 testing machines (malfunctioning)
Contextual	Facilitators	Short distance Less costs to clinic
	Barriers	Long distance high transport cost

### Results overview

While we identified both barriers and facilitators at all levels and reported by all respondents, we found that individual-level and health system-level factors reported by both People living with HIV (PLHIV) and healthcare providers were particularly prominent. With regard to the individual-level factors, most of the HIV positive individuals mentioned stigma as an important barrier. During the FGDs and IDIs with the PLHIV, the participants kept steering the discussions toward stigma related issues. With regard to the health system factors, both the PLHIV and the healthcare providers emphasized over-crowding and long waiting times at the centers.

We also noticed that in discussing the health system level in one of the sites, while the PLHIV mentioned that there were occasional medication stock-outs, the healthcare providers did not agree that this happens.

### Individual level

The individual level includes issues related to the individual’s knowledge, attitudes, feelings, experiences and behaviors towards linkage to care or accessing health care services.

### Facilitators to linkage to care

Three themes were identified as facilitators to linkage to care at the individual level: being sick or having symptoms at the time of diagnosis, disclosing one’s status to someone and social and moral support from relatives and from other PLHIV:

#### Individuals being sick at the time of diagnosis

The individual’s perception of their health status, and specifically being sick at or around the time of diagnosis, was reported (by PLHIV) to influence linkage to care positively. This is illustrated in the following excerpts:

“Initially, I didn’t think it was necessary to go [to the hospital] but one day I had a fever. I felt my hands and legs not working at all. I thought I am going to die. I asked my sister to take me to the hospital for medication” [IDI-Client_3_].

“They stay at home until they are seriously sick is when they go to the hospital for registration” [FGD_7_].

#### Individuals disclosing their status to someone

Respondents cited disclosure of seropositive status as an important factor in facilitating linkage to HIV care in HIV-positive individuals. For example, one participant reported:

“Most of my relatives know my status after since I told them. They support me. Sometimes they escort me to the clinic” [IDI-Client_3_].

#### Social and moral support from family members/relatives and from other PLHIV

Social and moral support from family and relatives were reported to enhance linkage behaviors of HIV-positive individuals in a number of the focus groups and individual interviews:

“I started suffering so I decided to ask my elder brother to escort me to do the test [HIV] and I was found HIV positive” [FGD_3_].

“My sister encouraged me, she said it not the end of the world, I will be okay when I start medication, and there are so many people who are in this [HIV] situation” [IDI-Client_8_].

“At our place Lusungo, we have formed the HIV support group which we meet every 15 of the month to discuss issues and encourage each other… we contribute little money for emergency …like if someone does not have transport fare we give” [FGD_2_].

### Individual level barriers to linkage to care

Six themes were salient as barriers to linkage to care from this sub-theme: fear of stigma, denial and being asymptomatic at the time of diagnosis, lack of understanding of the importance of being in care, lack of disclosure, belief in witchcraft and traditional treatment and spiritual beliefs.

#### Fear of stigma

Stigma related to attending HIV care clinics or CTCs was the most frequent factor described by the PLHIV and health care providers as a barrier to being registered (linked) into HIV care and treatment. This was captured in statements such as the following:

“They do not like going to the hospital; some fear that other people will see them at the clinic and know that they are infected” [FGD_1_].

“Most of them is because they fear that people will see them at CTC and start pointing fingers” [IDI-HCP_2_].

#### Denial and being asymptomatic

Denial of test results was reported as a prominent barrier to linkage to care among individuals who had tested positive for HIV:

“I was pregnant and I went to the clinic for antenatal care. They [clinicians] tested my blood and told that I am infected with HIV. I did not believe it because I was not sick and had no other health problem apart from being pregnant” [IDI-Client_9_].

“Some clients do not believe they are infected because they are healthy” [IDI-HCP_7_].

#### Poor health literacy (lack of understanding of the importance of being in care)

Poor health literacy in the form of lack of understanding of the importance of being in care was a hindrance to linkage to care. The issue of poor health literacy was mostly reported by the health care providers. The patients did not particularly allude to issues related to poor health literacy. Two examples are shown by the following quotes:

“Maybe education is still low, they do not understand that is importance to start ART care while you are still strong than when you are very weak” [IDI-HCP_9_]

“Also some people are just ignorant especially those who do not have any symptoms, they do not see the importance of being in care if they are not sick.” [IDI-HCP_5_].

#### Lack or fear of disclosure

Both PLHIV and the health care providers identified the lack or fear of disclosure as an important barrier to linkage to care. Participants revealed that HIV positive individuals sometimes fail to disclose their HIV status to their significant others for fear of consequences such as intimate partner violence and divorce. This is captured in the following statements:

“Many women are facing problems when disclosing HIV status to the men and some lead to divorce” [FGD_3_].

“They do not tell their partners, so it becomes difficult for them to come to the clinic and they come secretively” [IDI-HCP_13_].

#### Belief in witchcraft and traditional treatment

The reports of some of the HIV-positive individuals suggest that belief in witchcraft is a challenge to linkage to care among some HIV-positive individuals in rural areas. These individuals believed that they are sick due to witch craft (someone has bewitched them) so treatment at the hospital was not an option for them to get better. These experiences were mostly shared by the PLHIV

“When I started getting sick, my in-law took me to a traditional healer, he said I am bewitched. He [traditional healer] started treating me with different herbs, some for drinking and others for bathing. I wasn’t getting any better so I told him; I want to go to the hospital” [FGD _2_].

“He [traditional healer] said it was one of my neighbors in the market who is jealous of me. He [the neighbor] had put something in my store that is causing us to suffer from unknown diseases” [IDI-Client _2_].

#### Spiritual belief

It was also reported by some PLHIV as well as by some health care providers that certain spiritual beliefs sometimes had a negative impact on linkage to care Respondents reported that some people believed that prayers and usage of holy water can heal HIV, thus influencing their decision to link to HIV care. This was captured in most focus group discussions with the patients, and some of the key informant interviews with the health care providers support this point of view:

“You know when there is a problem you become worried so you can come here [clinic] or try other places. They say they pray for you and you will be healed” [FGD _4_].

“Some patients go to the new churches. The pastors in those churches they pray for them and give them holy water to take and they tell them you will be healed” [IDI-HCP _3_].

### Health care provider level

These are factors related to the relationship between the care providers and the PLHIV as well as to the behaviors of the health care providers. The facilitators and barriers under this level were reported by the PLHIV in the focus group discussion and individual interviews. Health care providers placed less emphasis on this level, particularly in relation to barriers.

### Health care provider facilitators to linkage to care

Two themes were identified as facilitators for linkage to care under this theme: Support or encouragement from health care providers and good patient/staff relationship.

#### Support or encouragement from health care providers

There were similar testimonies from both PLHIV and health care providers espousing that support and encouragement from care providers facilitated the registration (linkage) of HIV-positive individuals in the CTC. The role of support and encouragement is captured in these excerpts:

“Frankly speaking, the health providers here treat us nicely. They explained to me step-by-step on how to use drugs. I thank them. It is two weeks now since I started the drugs. They are providing good services” [FGD_3_].

“They [the nurses] even gave me the drugs for preventing chest infection and another disease.” [IDI-Client_6_].

#### Good patient-health care provider relationship

A patient who took part in the focus group discussions indicates that a good relationship and service delivery from the health care providers could encourage patients to link to care. A good relationship between the health care provider and the patient was reported to enhance linkage and continuity in HIV care as reported in some focus group discussions:

“The nurses are very polite and helping us a lot” [FGD_1_].

“Generally the service providers are treating us well; we do not have any complaint” [FGD_4_].

### Health care provider barriers to linkage to care

Negative healthcare provider attitude and use of abusive language were reported by the HIV positive individuals, particularly in one FGD and one IDI. On a follow up visit, a healthcare provider in the site in question claimed that this is related to a shortage of staff at the site: sometimes staff are overwhelmed by responsibilities and work overload, so the patients feel neglected.

#### Poor health care provider attitude and use of abusive language

Some participants reported that negative healthcare provider attitudes and the use of disrespectful language and shouting by some of the health care providers was an important barrier to linkage to care. It was also reported that patients dropped out of care when mistreated at the clinic. Participants in two of the focus group discussions expressed this in the following statements:

“When we reach here [clinic], they look at us like we are not normal human beings, they discriminate against us. They tell us to come very early but you see they start attending to us at 01:00 in the afternoon and sometimes you end up not getting the drugs” [FGD_2_].

“Truly, shouting can contribute so much, it hurts. You think I am sick then doctor barks or shouts at me, so they decide to stop coming” [FGD_6_].

### Health system level

Factors related to the health systems and to HIV care program implementation in the facility may include the organization of the health care services, leadership, resource availability (including human resources) and access to health promoting services like home based care or HIV support groups at clinics.

### Health system facilitators for linkage to care

Two themes were identified as facilitators to linkage to care under this theme: availability of referral procedures i.e. provision of a referral letter/referral form, and the existence of a well-organized clinic system with HIV testing service and HIV care services (a “one stop shop”), as well as possibilities of same day registration.

#### Availability of referral procedures

Most of the reports attesting to the availability of referral process were provided by the healthcare providers. Some of the PLHIV narratives support the reports of the healthcare providers. Providing a referral letter/form to enable the registration process of individuals at the CTCs was reported to facilitate linkage to HIV care and treatment. The referral letter is an important document given to the HIV positive individuals to confirm their seropositive status and facilitate registration into a CTC. The role of the referral letter in the care linkage process is captured in these statements below:

“If the client is positive, I refer him/her to CTC with this form (referral form) we have to make sure they are registered in the book” [IDI-HCP_1_].

“We refer the client we give him/her a referral letter with CD4 results print out and we also do HIV staging so that the CTC clinician can decide on how to continue” [IDI-HCP_7_].

It was also revealed that in some of the sites the peer health educator escorted the newly diagnosed individual to the clinic.

“I sometimes take the letter from here (Testing site) and I accompany the person to the clinic at the [District] hospital, I take them through all steps” [FGD _1_].

#### Service design or clinic services organization

Both the healthcare providers and the PLHIV agreed that the availability of HIV testing, CD4 counts and HIV care and treatment services within the same facility, organized to provide a complementary service, enabled linkage to care. This provides the possibility for individuals who test positive to be registered, thus linked to care at the CTC on the same day.

“Normally when I find a positive client, I go to the next room where we keep the documents for registration and the CTC cards, so I register him/her in the CTC register and give them a CTC clinic number with the treatment card” [IDI-HCP_1_].

“He [the care provider] gave me the letter and asked me to go to room number 10, show this [registration card] to the nurse. They gave me a card then I went into another room to give blood for CD4” [FGD _2_].

### Health system barriers to linkage to care

Five themes were identified as barriers to linkage to care within the health system umbrella. These include poorly organized clinic procedures and visits schedules, clinic overcrowding, long waiting times at the clinic, inadequate resources including CTCs, and shortage of staff.

#### Disorganized clinic procedures and visit schedules

While it was reported by the PLHIV that healthcare facilities that can provide integrated services of testing and treatment/care facilitated linkage to care, narratives from both PLHIV and healthcare providers indicate that the services might not be available on the same day. Some of the healthcare facilities had a schedule of when testing takes place and when registration for treatment and care occurred. This required patients to make multiple visits to the facility. For example, an individual testing on Friday morning will be given a referral letter to come the next Tuesday for registration, and then they will be told to come on Wednesday or Friday for CD4 testing and then given another appointment to get their CD4 results.

“We do CD4 testing on Wednesdays and Fridays, the other days is for in-patients’ tests. So if it is Wednesday and the client comes early, they go directly to the CD4 testing section and they are given a date to come for results normally. This could be in about three or four days’ time” [IDI-HCP_10_].

“He gave me some papers (referral form) to go with and show the nurse at the CTC. I went and the nurse said I should come on Tuesday for registration” [IDI-Client_2_].

#### Clinic overcrowding

Reports from both patients and healthcare providers indicated that clinic overcrowding could constitute a barrier to linkage to care. When patients found a huge crowd of people when they arrived at the clinic, they were tempted to return home as explained by a healthcare provider. This could lead to the patient failing to link to care timeously. The discussions among the PLHIV in a focus group supported this perspective.

“So even when you tell a client to go and join the queue for registration, he says ‘there are too many people, I will come tomorrow’ and that is it. They disappear. We don’t know whether they go to other clinics or what happens to them” [IDI-HCP_9_].

“For the matter of staying a long time it is because we are so many, therefore, we take a lot of time” [FGD_1_].

#### Long waiting times at the clinic

This was among the frequently mentioned barriers by both PLHIV and healthcare providers. Overcrowding in the CTCs and low numbers of healthcare providers were seen as leading to long waiting times for patients and hence a potential barrier to linkage to care.

“Patients wait for services for a very long time because the same staff has to go in the wards to assist, and then come here again for HIV client. For example, on Wednesday when they come for adherence treatment classes we can only start with them after 12 noon, and they always complain” [IDI-HCP_6_].

“We wait for services for a very long time and the waiting place is open when it is raining, we suffer a lot” [IDI-Client_1_].

#### Inadequate resources and equipment

Shortage of resources such as HIV care centres, staff and CD4 testing machines were identified as possible barriers to linkage in care. Statements from both healthcare providers and HIV-positive individuals suggest how this resource shortage could interfere with linkage of HIV-positive individuals into HIV care.

“There is a severe shortage of staff here; clients wait for CTC services for hours before they are attended to. We are only two nurses and one doctor per shift if one of them is sick or on leave, patients wait up to 4 o'clock in the evening” [IDI-HCP_6_].

“The problem is, we depend on only this hospital for all people in Chunya, people from Lupa tingatinga and all other villages…and Chunya is big” [FGD_4_].

In one district, the recurrent breakdown of the CD4 machine was mention as a serious concern:

“Also, the recurrent problem is with the CD4 machine [breakdown]. Maybe the government can help us by buying a new machine, even two we are so many here who need the service” [IDI-Client_1_].

“Most of the patients there are delayed due to CD4. This is the only site with a working CD4 machine. We receive patients from Sumbawanga even Nakonde from Zambia because this service is not available in their areas” [IDI-HCP_14_].

### Contextual level

Contextual factors refer to matters associated with access to, and affordability of, ART treatment and care services, and may also include social and cultural factors beyond individual beliefs concerning HIV infection. Depending on how far the individual lives from the clinic, the themes identified under this category could be either a facilitating factor or a barrier. Those who live close to the clinic did not identify access to the clinic facility as a possible barrier to linkage to care. Conversely, those who live further from care facility saw distance and transport cost to the facility as a potential challenge to linkage to care.

### Contextual facilitators of linkage to care

#### Proximity and low travel cost to the clinic

Proximity and hence lower transport costs to the clinic was reported by the PLHIV to facilitate linkage to care.

“I do not live very far from here, I just get a boda-boda [hired motorbike] and I pay only one thousand [less than half a dollar] and I can even walk if I want to” [IDI-Client_6_].

“For me, distance is not a problem, I walk for about ten minutes and am here, sometimes I send my daughter to bring the card for me and put it in the box while am still at home, when I come here I just wait for my turn to take medication” [FGD_7_].

### Contextual barriers to linkage to care

#### Long distance and high transport cost to CTCs (transport challenges)

Long distance to the clinic and the high cost of transport was reported by the participants (especially in two districts, Chunya and Mbozi), thus is identified as a deterring factor to linkage to HIV care and treatment. This is what some of the PLHIV and the care providers said:

“We refer our clients to either … hospital [about 10-15 Km] to… the health center also about same distance. They say ‘I do not have bus fare for now” [IDI-HCP_11_].

“Transport is very costly. Sometimes, I borrow money from friends and sometimes I come with my bicycle but I do not have the energy to ride for a long distance” [IDI-Client_2_].

“Binti-manyanga is far, it is about 100 kilometers from here and the cost of transport is very high about 28000 to 30000 [about $14] per trip” [FGD_4_].

## Discussion

**Fig. 1:**
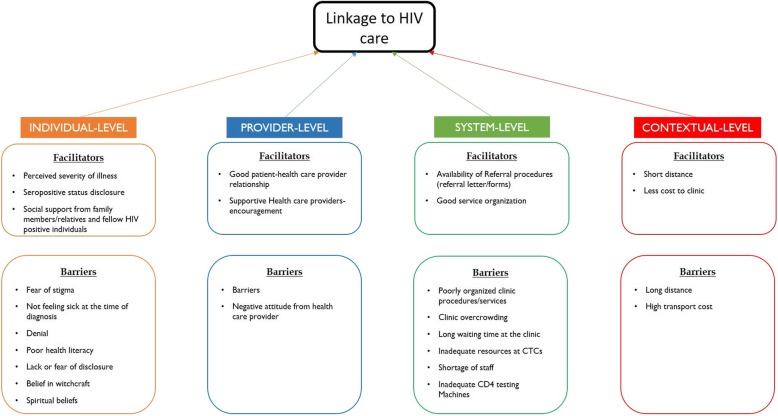
Conceptualizing the factors influencing linkage to HIV care in Mbeya, Tanzania

The study sought to explore the facilitators and barriers to linkage into HIV care after testing HIV positive in the rural settings of Mbeya region from both facility-based and mobile/outreach sites, aiming at identifying challenges/gaps and informing implementation strategies needed for strengthening health services and care for individuals diagnosed with HIV. The literature on linkage to care indicates that while many studies have explored and described barriers to linkage to HIV treatment and in various settings, fewer studies have looked at the facilitating factors and to a lesser extent exploring both together. In this study, multiple factors were identified to influence linkage to care in newly HIV positive diagnosed individuals and we identified these factors under four levels, namely individual, healthcare provider, health system and contextual levels (Fig. 1).

Based on the findings of this study, as indicated in Fig. 1, access and linkage to care plays out at different levels, however, the individual-level and health system-levels barriers were most prominent. At the individual level, HIV positive individuals cited stigma as a very important barrier to linkage to care. This underlines the importance of scaling up of educational programs and community HIV-support groups. At the health system level, clinic overcrowding and long waiting times were emphasized by both the HIV-positive individuals and the healthcare providers. These findings are in line with similar studies in Sub Saharan African countries.

### Individual level

At the individual level, the study revealed that having symptoms of disease (being sick at the time of diagnosis), the disclosure of HIV status, and support from family/relatives were facilitators to linkage to care. This finding is supported by the HIV positive individuals and healthcare providers who participated in the qualitative study. Social and moral support from the HIV-positive individuals’ families and spouses, although not identified in the quantitative data as significant to linkage to care were salient in the qualitative inquiry. This result corresponds to other similar studies on linkage to care [34–37].

In this study, the most frequently reported barriers to linkage into HIV care include fear of stigma related to HIV, lack of disclosure, being asymptomatic at the time of diagnosis and denial. However, belief in witchcraft and belief in spiritual healing were reported to hinder linkage into HIV care. Similar findings were identified in other studies on linkage to HIV care in Sub-Saharan countries [19, 21, 36, 38, 39]. This emphasizes the importance to empower communities, households and support multiple stakeholders in increasing community HIV/AIDS awareness and establishment of HIV support groups in communities to discussing issues related to stigma and access to HIV care [24, 40].

### Healthcare provider level

We found that healthcare providers’ moral and practical support and good patient-staff relationships enhance linkage to HIV care. Conversely, the perceived experience of negative provider attitudes, lack of respect and/or use of abusive language from healthcare providers to patients was reported by the study participants to impede successful linkage to HIV care. Other studies report corresponding results [10, 19, 21, 41]. However, in this study, the healthcare providers in some instances suggested that negative relationships with clients are related to a shortage of staff at the site and high workloads. Existing literature indicates that basic resources, effective management and supportive supervision issues need to be addressed within a health system in order for the patient-centered care of high interpersonal quality to become a priority for health care providers [42, 43].

### Health system level

At the health system level, we found the availability of good referral procedures and well-organized clinics services to be important facilitators for linkage to HIV care. Elul et al., (2014) in their study on enhancing linkage to care reported that availability of CD4 testing at the point of care and a short message service (SMS) reminder of appointment to patients was a facilitator to linkage. We also found that in some clinic settings, testing and linkage to care were performed on the same day (same day registration) and sometime CD4 test was also done on the same day.

Healthcare providers indicated that the mobile clinic staff took measures to promote linkage to care. For instance, one of the healthcare providers running the mobile clinics reported that they situated their mobile laboratory near a health facility such as a hospital, health center or dispensary to link their HIV and TB clients to the existing system for further care. It was also reported by the healthcare provider from mobile/outreach services that the peer educator, usually after counseling and testing, escorts the newly diagnosed individuals to the care center to ensure linkage. By adopting the above-mentioned strategies, the linkage to care rates from the mobile testing individual could be improved; these findings suggest that the disadvantages of testing outside of a multi-service facility are not overcome even by highly motivated and proactive staff action.

Barriers to linkage in care at health system level included poorly organized clinic procedures and visits schedules, overcrowding and long waiting time and lack of resources including staff and equipment at the clinic were salient barriers to successful linkage into HIV care. While some studies have indicated that having HIV testing services and HIV care on the same spot improves rates of linkage to care and ART coverage [20, 21], the integration of these services, if not well coordinated and organized could also constitute a barrier to linkage to care. These factors were similar to some of the health system barriers reported in other HIV care linkage studies [6, 17, 39, 44]. However, the qualitative findings reported here, suggest that even poorly integrated « one stop shops » are preferable to multiple visits to separate facilities.

### Contextual level

The context level factors that were identified in this study were related to the distance to the healthcare facility and the cost of transport. For respondents living in close proximity to the clinic, distance and cost were not a challenge, however, it was a prominent challenge to respondents who lived further than 10 km. However, our earlier analysis of a larger quantitative dataset indicated that there was no significant difference between the rates of linkage to care for HIV-positive individuals who lived less than 10kms from the healthcare facility compared to those who lived more than 10kms from the healthcare facility [23]. Other barriers identified by the study participants at the individual, provider and institutional levels could have influenced the decisions not to register for HIV treatment and care more than the influence of the distance. Our findings also suggest that both respondents extrapolate from their own personal experience, since participants for whom distance was a personal barrier reported it as such, while those who lived close to a facility did not identify that this could be a potential barrier.

A number of studies have also reported the influence of long distances and transport costs on linkage to care behaviors of individuals diagnosed with HIV [3, 15, 44, 45]. Furthermore, some of the HIV-positive individuals reported that short distances to the clinic and low transport cost improved the rate of linkage to care.

Social and moral support from family members, friends, neighbors and significant others were reported to facilitate linkage and retention in HIV care, echoing findings reported by Genberg et al. [20] and Gerdts et al. [46]. A possible mediator between disclosure of HIV status and early linkage is the perceived social and moral support that the HIV-positive individuals receive from their significant others after disclosure.

### Strengths and limitations

One of the strengths of this study is that various stakeholders (Healthcare providers, and HIV-positive individuals), different data collection methods (FGDs and IDIs) and multiple sites (16 sites) were used to elicit relevant information. By triangulating the data source, collection methods and sites, we improved on the trustworthiness of the study.

However, a limitation of the study is that, while we explored the challenges and facilitators for linkage to care, we only interviewed individuals who were already under the care umbrella. We did not have access to those individuals who tested HIV positive but were not registered in care. In addition, we did not delineate the responses of those who tested in the mobile sites from those who tested at the facilities because both groups were entering care into the health facility-based sites. While conducting this study, it was identified that poor leadership and management at some of the facilities were responsible for poor linkage to care behaviors. Although this study focused on the factors influencing linkage to care, it did not focus in particular on the broader health system issues like health management and leadership. This may require further investigation to address the issues around leadership and health systems management.

## Conclusion

Linkage to HIV care is an important step towards proper lifelong management of HIV infection. The findings of this study indicate that while there are many barriers to linkage to care, facilitating factors are also explicitly identified by patients and by healthcare providers. We found that individual, healthcare provider, health system and contextual factors might all influence linkage into care, but that in this study setting, the individual-level and health system-level factors were the most prominent factors influencing access and linkage to care positively and negatively. In most cases the PLHIV and health care providers’ opinion were congruent except at the health care provider level where it was the PLHIV who shared their experience of less than optimal care. These findings emphasize the need for further problem –focused and action oriented strategies addressing individual level factors, notably stigma, and health system factors, notably under-resourced and poorly coordinated facilities. These challenges and the respective strategies are particularly important from the perspective of both patients and providers.

### Implication for policy and practice

This study was undertaken in part to inform future interventions strategies for hard-to-reach, low-income settings with moderately high HIV prevalence rates. Interventions to achieve optimal coverage in light of adopting the ‘Test and Treat’ model of HIV treatment and care recommended by the World Health Organization must address at least the individual issues around fear of stigma, not feeling sick at the time of diagnosis, denial, poor health literacy, lack or fear of disclosure, beliefs in witch craft and concomitant medications. If linkage to care is to be improved, health system level factors in particular must be addressed, notably poor clinic procedures and visit schedules, clinic over-crowding, long waiting times at the clinic, and adequacy of health workers and CD4 testing machines. Among interventions that could be implemented to address some of the challenges of linkage to care are improved counseling strategies and the establishment of HIV support groups and community interaction programs to discuss and address HIV stigma-related matters in the community. At the health system level, continued scale-up of HIV testing and treatment, removing barriers to access to HIV care services, and improving availability of human and other resources are pertinent. In addition to this, improving HIV care clinic procedures for the provision of better quality of care and treatment services to PLHIV is needed. Implementation of interventions aiming at actively strengthening the identified facilitating factors – which largely mirror the barriers - may be particularly promising in enhancing the capabilities of the government health systems, delivery of services and health care providers in the rural settings.
